# Transcriptome analysis revealed that a quorum sensing system regulates the transfer of the pAt megaplasmid in *Agrobacterium tumefaciens*

**DOI:** 10.1186/s12864-016-3007-5

**Published:** 2016-08-20

**Authors:** Nadia Mhedbi-Hajri, Noura Yahiaoui, Samuel Mondy, Nathalie Hue, Franck Pélissier, Denis Faure, Yves Dessaux

**Affiliations:** 1 Institut for integrative biology of the cell, CEA, CNRS, Université Paris-sud, Université Paris-Saclay, 91198 Gif sur Yvette, CEDEX France; 2 Institut de chimie des substances naturelles, CNRS, Avenue de la terrasse, 91198 Gif sur Yvette, France; 3 Present address: CIRAD, 7 chemin de l’IRAT, ligne Paradis, 97410 Saint Pierre de la Réunion, France; 4 Present address: UMR1347 Agroécologie, INRA, Centre de Dijon, 17 rue Sully, BP 86510, 21065 Dijon, CEDEX France

**Keywords:** *Agrobacterium*, At plasmid, Transcriptomics, Conjugation, T4SS, Dtr system, Acylhomoserine lactone, Quorum sensing

## Abstract

**Background:**

*Agrobacterium tumefaciens* strain P4 is atypical, as the strain is not pathogenic and produces a for this species unusual quorum sensing signal, identified as *N*-(3-hydroxy-octanoyl)-homoserine lactone (3OH,C8-HSL).

**Results:**

By sequence analysis and cloning, a functional *luxI*-like gene, named *cinI*, has been identified on the At plasmid of *A. tumefaciens* strain P4. Insertion mutagenesis in the *cinI* gene and transcriptome analyses permitted the identification of 32 *cinI*-regulated genes in this strain, most of them encoding proteins responsible for the conjugative transfer of pAtP4. Among these genes were the *avhB* genes that encode a type 4 secretion system (T4SS) involved in the formation of the conjugation apparatus, the *tra* genes that encode the DNA transfer and replication (Dtr) machinery and *cinI* and two *luxR* orthologs. These last two genes, *cinR* and *cinX*, exhibit an unusual organization, with the *cinI* gene surrounded by the two *luxR* orthologs. Conjugation experiments confirmed that the conjugative transfer of pAtP4 is regulated by 3OH,C8-HSL. Root colonization experiments indicated that the quorum sensing regulation of the conjugation of the pAtP4 does not confer a gain or a loss of fitness to the bacterial host in the tomato plant rhizosphere.

**Conclusion:**

This work is the first identification of the occurrence of a quorum sensing regulation of the pAt conjugation phenomenon in *Agrobacterium*.

**Electronic supplementary material:**

The online version of this article (doi:10.1186/s12864-016-3007-5) contains supplementary material, which is available to authorized users.

## Background

Members of the species *Agrobacterium tumefaciens* are plant pathogens, inducing the formation of tumors mostly at the crown of dicotyledonous plant species. The main pathogenic element of the bacteria is the tumor-inducing (Ti) plasmid, a fragment of which, the T-DNA, is transferred from the bacteria to the plant cell upon infection (for reviews, see [1–4]). The expression of T-DNA genes in the plant leads to the formation of the tumor and to the synthesis, by the tumor cells, of specific molecules, the opines. Opines are instrumental in the ecological niche constituted by the tumor [5]. First, they are used as growth substrates by the tumor-inducing *Agrobacterium* strains via the products of genes located on the Ti plasmid. Second, some of them also induce the conjugative transfer of the Ti plasmid (for reviews, see [6, 7]).

In the *A. tumefaciens* reference strain C58 (genomospecies G8), the genome consists of two chromosomes, a circular one (ca. 2.8 Mbp) and a linear one (ca. 2.1 Mbp), plus an At plasmid (ca. 540 kbp) and a Ti plasmid (ca. 214 kbp [8, 9]). This latter plasmid bears most of the genetic determinants involved in the induction of plant tumors that are characterized by the presence of opines of the nopaline and agrocinopine families. Nopaline is an arginine derivative while the agrocinopines (A and B) are arabinose phosphate derivatives (for a review, see [6]). These last two opines induce the conjugative transfer of the C58 Ti plasmid in conjunction with a quorum sensing (QS) regulatory pathway [10–14] (for reviews, see [7, 15]). In bacteria, QS couples gene expression with bacterial cell concentration. It involves the production and sensing, by members of a bacterial population, of diffusible signals, the concentration of which mimics that of the bacterial cells. Once a threshold cell density is reached (*i.e.* a quorum), the concentration of the signals permits their perception and the subsequent activation or repression of the transcription of QS-regulated genes (for reviews, see [16–18]). In *A. tumefaciens*, the QS signal molecule that controls the conjugative transfer of the Ti plasmid belongs to the most common signal class in Proteobacteria, *i.e.* the *N*-acyl homoserine lactone (AHL) class; it is *N*-(3-oxo-octanoyl)-L-homoserine lactone (3O,C8-HSL; [10, 12]). AHLs are generally synthesized by the prodcucts of *luxI* and *luxI*-like genes (for reviews, see [16–18]).

In strain C58, the genetic determinants involved in the conjugative transfer of Ti plasmid are induced by the agrocinopines, which bind the AccR regulator encoded by the *accR* gene (Additional file 1). This releases the repression of transcription of the *accABCDEFG* operon encoding the transport and degradation of the agrocinopines A and B. Bound to agrocinopines, the AccR regulator also derepresses the expression of the *arc* operon. Among the *arc* genes is *traR*, a member of the *luxR* family that encodes the QS regulator TraR. This latter regulator binds the QS signal molecule 3O,C8-HSL, dimerizes, and activates the transcription of the *tra* and *trb* operons involved in the conjugative transfer of the Ti plasmid, and to a lesser extent that of the *rep* operon involved in the replication of the Ti plasmid [19] (for reviews, see [7, 20, 21]). This complex regulatory system implies that C58 Ti plasmid conjugation can only occur at high donor cell density and in agrocinopine-rich environments, allowing the efficient dissemination of the main pathogenic element of the bacteria in tumors.

In contrast with the wealth of data dealing with the Ti plasmid, little is known about the At plasmid in spite of the fact that it is often found in natural isolates of *Agrobacterium spp.* and can represent up to ca. 10 % of the bacterial genome. The At plasmid can sustain some large deletions (25 to 30 % of the plasmid size) that are associated with a significantly reduced carrying cost to the cell [22]. Known functions of this plasmid are scarce. One is the utilization of gamma-butyrolactone and gamma hydroxy-butyrate via the products of the *blcABC* (also known as *attKLM*) operon [23]. The last gene of this operon, *blcC* (aka *attM*) encodes the lactonase that hydrolyses gamma butyrolactone, as well as numerous QS signal molecules of the AHL class [24]. The At plasmid also harbor genes involved in the degradation of Amadori compounds such as deoxyfructosyl-glutamate and deoxyfructosyl-glutamine, that are commonly found in decaying plant material [25]. At plasmids are also conjugative plasmids. Sequence analyses of the C58 genome have revealed the occurrence of a type 4 secretion system (T4SS), termed AvhB, on pAtC58 that is highly similar to the VirB system of *Bartonella henselae* [26]. The pAtC58 T4SS (T4SS_pAt_) also shares some common traits with two previously identified T4SS of *Agrobacterium*, *i.e.* the one encoded by the virulence (*vir*) genes of the Ti plasmid (involved in T-DNA transfer to the plant cells and thus in disease induction) and the one encoded by the *trb* genes (T4SS_pTi_, involved in the conjugative transfer of this plasmid [27]). Interestingly, a recent study revealed that also the T4SS_pAt_ of strain C58 is regulated by the master regulator AccR encoded by the Ti plasmid *accR* gene [28], a feature that favors the co-transfer of both *Agrobacterium* megaplasmids in the agrocinopine-rich environment of the tumor.

*Agrobacterium* strain P4 was previously isolated from a plant rhizosphere [29]. This strain exhibited the capacity to synthesize a QS signal of the AHL class. Strain P4 is non-pathogenic and in agreement with this, no Ti plasmid was observed following the complete sequencing of its genome [30]. We report here the structure of the AHL produced by P4, we investigated the possibility that this AHL is indeed a QS signal and we identified the relevant determinants in QS. Moreover, we report the occurrence of a QS regulation of the expression of the T4SS_pAt_ in strain P4.

## Methods

### Bacterial strains and growth conditions

The bacterial strains and plasmids used in this study are described in Table 1. *Agrobacterium* strains were routinely cultivated at 28 °C in modified Luria Bertani medium (LBm [31]), tryptone-yeast medium (TY: bactotryptone 5 g/L, yeast extract 3 g/L) or in *Agrobacterium* broth (AB) minimal medium [32] supplemented with ammonium chloride (1 g/L) and a carbon source (mannitol or glucose 2 g/L, for AB mannitol and AB glucose, respectively). When necessary, media were solidified using bacto-agar (Becton-Dickinson), 15 g/L. *Escherichia coli* cells were routinely cultivated at 37 °C in LBm. Media were supplemented with appropriate antibiotics that were added at the following final concentrations: rifampicin 100 mg/L, streptomycin 250 mg/L, gentamicin 25 mg/L, carbenicillin 100 mg/L and ampicillin 50 mg/L.

**Table 1:** Bacterial strains and plasmids used in this study

Strain or Plasmid	Relevant genotype or characteristics	Reference or source
Strains		
P4	*Agrobacterium tumefaciens*. Isolated from tobacco rhizosphere (La Côte Saint André, France)	Mondy *et al.*, 2013
P4cinI	*A. tumefaciens*; Gm^r a^, pAt*cinI*::Gm	This study
P4Rif	*A. tumefaciens*; Rif^r^	Laboratory collection
P4RifcinI	*A. tumefaciens*; Rif^r^, Gm^r^, pAt*cinI*::Gm	This study
P4RifNC	*A. tumefaciens*; Rif^r^, Gm^r^, pAt::Gm	This study
P4RifRIX	*A. tumefaciens*; Rif^r^, Gm^r^, pAtΔ*cinRcinIcinX*::Gm	This study
C58.00	*A. tumefaciens*; Rif^r^, Sm^r^, cured of the At and Ti plasmids	Laboratory collection
NT1(pZLR4)	*A. tumefaciens* ^b^ Gm^r^, NT1 derived from strain C58, carrying plasmid ZLR4; OC8-HSL biosensor	Cha *et al.*, 1998 ; Pipper *et al.*, 1993
DH5α	*Escherichia coli*; F, Φ 980dlacZ : M15, recA1, endA1, gyrA96, thi-1, hsdR17(rK–, mK+), supE44,relA1, deoR:(lacZYA–argF)U169	Hanahan, 1983
Plasmids		
pGEM®-T Easy	Amp^r^, high copy number	Promega Crop., Madison, WI

^a^abbreviations are as follows: Rif^r^, Sm^r^, Gm^r^, Amp^r^, resistant to rifampicin, streptomycin, gentamicin, and ampicillin, respectively

^b^This strain is also a derivative of strain C58

### Identification, extraction and determination of the structure of acyl-homoserine lactones

Preliminary tests to evaluate the production of AHLs were performed essentially as described earlier [33]. Aliquots of overnight culture supernatants, or 10 fold concentrated ethyl acetate extracts of culture supernatants of the assayed strains, were spotted onto TLC silica plate (Whatman ref. 4801-800). Both *Chromobacterium violaceum* strain CVO26 [34] and *Agrobacterium* strain NT1(pZLR4) [33] were used as bio-indicators. When necessary, TLC plates were first submitted to chromatography in a methanol/water solvent system (55/45; *v/v*).

For the determination of the structure of AHL, 500 mL of LBm medium inoculated with the P4 strain were centrifuged (10 min., 4000 *g*, 4 °C) and the supernatant serially filtered on 1.8, 1.2 and 0.45 μm ultrafilters under vacuum. The filtered supernatant was extracted with ethyl acetate (*v/v*). Ethyl acetate was rotary evaporated to dryness at 30 °C, and the extract resuspended in 100 % acetonitrile prior to UPLC MS/MS analysis. UPLC MS/MS was performed on a Waters Aquity UPLC-TQD apparatus driven by a MassLynx 4.1 software, and fitted with an Acquity HSS C18 column (2.1 mm × 50 mm; 1.8 μm). The temperature was set at 20 °C and the solvent flow at 0.6 mL/min. The solvent composition is provided in Additional file 2. Mass spectrometry was performed by electrospay ionization. The triple quadrupole analyzer was used in both MS and MS/MS modes with a *m/z* mass ranging from 50 to 800. The most sensitive analytical conditions were first determined using synthetic *N*-(octanoyl)-L-homoserine lactone (C8-HSL) and 3O,C8-HSL. Synthetic *N*-(3-hydroxy-octanoyl)-homoserine lactone (3OH,C8-HSL) was used as a standard in UPLC MS/MS analyses.

### Gene sequence analyses

Identification of ORFs, determination of gene organization and comparative genomic analyses of plasmids and chromosomal regions were performed at the NCBI web site (http://blast.ncbi.nlm.nih.gov/Blast.cgi), MicroScope plateform (https://www.genoscope.cns.fr/agc/microscope/home/index.php) and INRA AGMI*AL* [35] plateform (http://maiage.jouy.inra.fr/?q=fr/logiciels). In this latter case, the Artemis program was used. Sequence comparisons were performed using BLAST tools (blastn, blastp and tblastn) with default parameters. BLAST analyses were achieved on both the non-redundant nucleotide sequence collection (nr/nt) and whole-genome shotgun contigs (WGS) databases. Prediction of functional domains was achieved using InterPro [36]. Protein sequences were aligned using ClustalW [37]. Phylogenetic trees based on protein sequence analysis were constructed using the Neighbor-Joining method implemented in MEGA program version 5 [38]. Bootstrapping was performed with 1,000 replicates to assess confidence on the nodes.

### Cloning and mutagenesis

Plasmid extractions were performed with the QIAprep® Spin Miniprep Kit (Qiagen, GmbH). Restriction enzymes, T4 DNA ligase (Thermo Scientific, GmbH), and T4 DNA polymerase (Eurobio, France) were used according to manufacturer’s recommendations. PCR assays were performed in a final volume of 25 μL containing 1 X DNA polymerase buffer (provided by the manufacturer), dNTPs 2.5 mM, MgCl_2_ 2 mM, primers (Table 2) 1 μM each, DNA polymerase 0.5 unit, and 1 μL of boiled bacterial suspensions. PCR conditions were 5 min at 94 °C, followed by 35 cycles of 1 min at 94 °C, 1.5 min at 58 °C, and 2 min at 94 °C, and a final 10 min incubation at 75 °C phase.

**Table 2:** PCR primers used in this study

Targeted gene or region		Primer sequence^a^
P4 *cinI*	F :	GGCGCAATACCTCAATTGATTG
	R :	CATCATGGACGTCGTATCGATC
P4 pAt non coding-region (coord. 36817–37590^b^)	F :	CATCTCGTTGATCGCAACATGGTCGTT
	R :	GATTTGGATGCTTCAACCGAAGATTTATTTGC
P4 pAt non encoding-region (coord. 36950–37353^b^)	F :	CCGAAAGTAACATTTGATGCCCCAACTGAATTTT
	R :	ATAACTGACAGGCATGCGAAAGCCGTC
P4 *cinR cinI cinX*	F :	TGCTTGTCGTCTGCGCCGTGAGA
	R :	CTCAAACACCGCTGGAGCGAACTCA
P4 *avhB5* RT-qPCR	F:	CCAAGCAGATCGAGAGCAT
	R:	TTGTTGAGCGAGCCATAAAG
P4 *avhB11* RT-qPCR	F:	ACGCTTTCCAAAGCATTGAT
	R:	CGTGGTTCGGTTGTGAGATA
P4 AGROTU_05920 RT-qPCR	F:	TTGTTGAATGTATGCGGGTT
	R:	TATGCGCTGTGGAGATATGG
P4 *traA* RT-qPCR	F:	CGAGCTGTTCCTCACTACAGT
	R:	TTCAATCTTTCCGACAGCACC
P4 *cinR* RT-qPCR	F:	CGTCGGAGAGTTCGGTTATT
	R:	CCTGCAACAAGTCTGTCCC
P4 *cinI* (internal) RT-qPCR	F:	ATATGGGAAGGCACACGAAT
	R:	AAAGGAACCCAAACGCTCTA
P4 *cinX* RT-qPCR	F:	AATATCGGCAGGAGCATTTC
	R:	GCTCATGCTCAAGGTCAAAG
P4 *traG* RT-qPCR	F:	GATTTCGATTCGACCCACAT
	R:	AGCGGGCCTGTATATCTGAG
P4 *gyrB* RT-qPCR	F:	GATGGCAAGGCCTATGAAAC
	R:	CACTTCCAGAAGATCGGACA
P4 *blcR* RT-qPCR	F:	CGACATTCAACGACCATCTC
	R:	CAGCCGATGTAAACGACTTC

^a^all primer sequences are given in the 5′- > 3 orientations. *F* forward primer, *R* reverse primer

^b^coordinates on pAt P4 with respect to the position of the first and last base amplified by the primer couples

To mutagenize *cinI*, the Gm cassette from plasmid p34S-Gm [39] was inserted at the unique *Xba*I restriction site of a pGEM®-T Easy vector (Promega France) harboring a *ca*. 800 bp region of the *cinI* gene obtained by PCR amplification (Additional file 3). The same cassette was also inserted at the unique *Sph*I restriction site found in a 1240 bp non-coding region of plasmid At (located in between 36783 bp and 38021 bp coordinates), previously amplified and cloned into pGEM®-T Easy. The resulting plasmids, which are not replicative in *Agrobacterium*, were electroporated (2500 V, 125 Ω, 50 μF) into P4 and P4Rif strains. Double crossing-overs that led to a marker exchange were identified by carbenicillin sensivity and gentamicin resistance assays and verified by PCR using the appropriate primers. The *cinI* mutants and the pAt non-coding control mutant were named P4cinI and P4RifcinI (depending on the background) and P4RifNC, respectively.

Another mutant harboring a deletion in the region *cinR cinI cinX* was generated by homologous recombination of a DNA fragment that consisted of a Gm-cassette surrounded by 375 bp of the 3′ end of the *cinR* gene and 336 bp of the 5′ end of the *cinX* gene (Additional file 3). This DNA fragment was synthesized and cloned into the pMA-T vector at the unique *Sfi*I restriction site by the provider (Life Technologies, Carlsbad, USA). The resulting plasmid which is not replicative in *Agrobacterium* was electroporated (2500 V, 125 Ω, 50 μF) into the P4Rif strain. Double crossing-overs that led to a marker exchange were identified by carbenicillin sensitivity and gentamicin resistance assays and verified by PCR using appropriate primers. This mutant was named P4RifRIX.

### Transcriptomic and RT-PCR analyses

Overnight cultures of *A. tumefaciens* strains P4 and P4cinI were diluted to an OD_600nm_ = 0.1 in 50 mL AB mannitol. Cells were grown at 28 °C up to an OD_600nm_ = 0.7 and harvested by centrifugation (15 min, 3500 *g*, 4 °C). RNA was extracted using the NucleoSpin RNA II kit (Macherey-Nagel Ltd., Oensingen, Switzerland) and further treated using RNase-free DNAse (Ambion, Austin, TX, USA) according to manufacturer’s instructions to ensure the absence of any contaminating DNA. RNA concentration and purity were assessed using a nanodrop ND-100. RNA samples were used in transcriptomics, RT-PCR and RT-qPCR experiments.

For transcriptomic analyses (performed at the high throughput sequencing platform of the Institute for Integrative Biology of the Cell, Gif-sur-Yvette, France), RNA quality was re-evaluated on an Agilent Bioanalyzer 2100, using an RNA 6000 pico kit (Agilent Technologies, Santa Clara, USA). Total mRNA were purified from 1 μg total RNA using Ribo-Zero rRNA (bacteria) removal kit (Epicentre, Madison, USA), according to the manufacturer recommendations. Directionnal RNA-seq libraries were constructed using ScriptSeq V2 RNA-seq library preparation kit (Epicentre, Madison, USA), according to the manufacturer’s recommendations (with 14 PCR cycles). Library quality was assessed on an Agilent Bioanalyzer 2100, using an Agilent High Sensitivity DNA Kit. Libraries were sequenced on an Illumina Hiseq 1000 instrument, with a TruSeq SR Cluster Kit v3-cBot-HS (Illumina, San Diego, USA) and a TruSeq SBS v3-HS - 50 cycles Kit (Illumina, San Diego, USA), using a Single Read 50 bp recipe. Libraries were pooled in equimolar proportions and diluted to a final concentration of 12pM, according to Illumina recommandations. The generated data were demultiplexed by the high throughput sequencing platform of the Institute for Integrative Biology of the Cell (Gif-sur-Yvette, France) using CASAVA software suite (CASAVA-1.8.2; Illumina, San Diego, USA). The quality of the data was checked with the software FastQC 0.10.1 from Babraham Bioinformatics, Cambridge, UK). The Illumina 3′-adapter was trimmed using Cutadapt-1.2.1 (Python community), and only reads with a minimal length of 10 nucleotides were kept. After the trimming step, the data were mapped using TopHat2 (Center for Computational Biology, John Hopkins University, Baltimore, USA) with a seed length of 8 (default value) to the appropriate reference genome of P4 (including 3 scaffolds). Only unique reads were analyzed. Bam output files were sorted with Samtools (http://www.htslib.org/). HTSeq-count (EMBL, Heidelberg, Germany) was used to evaluate the number of reads by gene or by CDS. The DESEQ package [40] was used to analyze the differential expression of genes between the reference strain P4 and the *cinI* mutant.

Standard RT reactions were performed with 1 μg total RNA obtained as indicated above, using random hexamers as primers and the Revert Aid TM HM Inis First Strand cDNA Synthesis Kit (Fermentas) according to the manufacturer’s instructions. RT-qPCRs were performed in a Lightcycler® 480 II (Roche) apparatus. The data were processed using the 2^-ΔΔCT^ method. For all primer sets (Table 2), the similarities of amplification efficiencies were controlled. The expression of the following 8 pAtP4 genes was investigated: *avhB11*, *avhB5*, *traA*, *traG*, *cinR*, *cinI, cinX*, and AGROTU_05920. The internal control used was the regulator gene *blcR* (Atu5136), also located on the At plasmid.

### Measurement of bacterial doubling time

Strains P4Rif and P4RifcinI were cultivated at 28 °C during 40 h in AB mannitol and AB glucose and in the rich media LBm and TY. For each condition, five independent repeats were set up per strain. Bacterial growth was estimated by measuring the OD_600nm_ every 30 min using a Biosreen C microplate reader and according to the manufacturer’s recommendations (Growth Curves USA, Piscataway, NJ, USA). Generation times were calculated based on the OD_600nm_ from graphical plots of the growth curves. The experiment was repeated 5 times.

### Conjugation assays

Conjugation assays were performed under *in vitro* conditions. Overnight cultures in LBm of donor and recipient cells were mixed at an equal cell density (*i.e.* ratio 1:1). Twenty μL of the mix were subcultured into 180 μL of AB mannitol and incubated in microplates at 28 °C for 72 to 96 h with no shaking, if necessary in the presence of 100 nM AHL. Dilutions of these mixed cultures were spotted onto LBm agar media supplemented with the appropriate antibiotics to enumerate the different bacterial populations, *i.e* the donor, recipient, and At plasmid transconjugants. The verification of the presence of the At plasmid in a subset of randomly picked, putative transconjugants was performed by PCR with appropriate primers (Table 2). For each experiment, at least 8 independent repeat assays were performed, and the conjugation frequencies expressed per recipient cells.

### Plant colonization under bacterial competition assays

Tomato plants (*Solanum lycopersicum* cv. F1 hybrid Dona, Vilmorin, France) were grown in a greenhouse under long day conditions (16 hours light), controlled temperature (24 to 25 °C night and day), in an unsterilized horticultural soil and daily watered with unsterilized tap water. At the zero time point, the pots containing three-week old plants were soaked with the bacterial suspensions (strains P4RifNC and P4RifRIX mixed at an equal cell density, i.e. ratio 1:1; final OD_600nm_ = 0.1) until the maximum water retention capacity of the soils was reached. Nine plants were inoculated with the bacteria mixture and 3 samples consisting of 10 g of rhizospheric soil (defined as the soil fraction strongly adherent to the roots) were obtained at each time point, i.e. 1 day post inoculation (dpi), 7 dpi, and 21 dpi. The soil samples were resuspended in 30 mL sterile NaCl 0.8 % and vigorously vortexed for 30 s. Dilutions of these suspensions were plated onto AB mannitol agar supplemented with rifampicin and gentamicin to enumerate CFU after a 2 day incubation. PCR amplifications were performed using the appropriate primers (Table 2) to discriminate strain P4RifNC from P4RifRIX on a subset of 150 randomly picked, enumerated colonies per time point.

## Results

### *Agrobacterium* strain P4 produced *N*-(3-hydroxy-octanoyl)-homoserine lactone (3OH,C8-HSL) but not 3O,C8-HSL

**Fig. 1:**
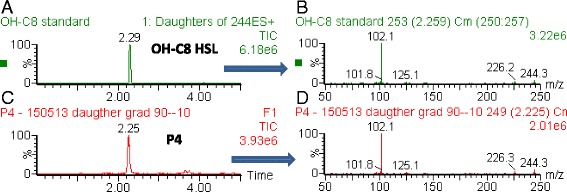
Identification of *N*-(3-hydroxy-octanoyl)-homoserine lactone (3OH,C8-HSL) in culture supernatants of strain P4. An ethyl acetate extract of a spend culture supernatant was concentrated, resuspended in 100 % acetonitrile and analyzed by UPLC MS/MS. Panel **a**: elution profile of synthetic 3OH,C8-HSL. Panel **b**: mass spectra of synthetic 3OH,C8-HSL. The m/z values 244, 226 and 102 correspond to the protonated forms of 3OH,C8-HSL (243 + 1), the dehydrated 3OH,C8-HSL (225 + 1) and the characteristic amine substituted lactone cycle (101 + 1), respectively. Panel **c**: elution profile of the concentrated culture supernatant of strain P4. The major peak migrates as the one of synthetic 3OH,C8-HSL. Panel **d**: mass spectrum of the major peak observed in the concentrated culture supernatant of strain P4. The m/z values and species are identical to those observed for synthetic 3OH,C8-HSL

Strain P4 was identified as one of the environmental strains that activates a beta-galactosidase activity in *A. tumefaciens* strain NT1(pZLR4) [29], a QS bioindicator that responds to long chain AHLs [41]. The production of short chain AHLs was assessed using the *C. violaceum* bio-indicator strain CVO26 and found to be undetectable. Ethyl acetate supernatant extracts were obtained from spent culture media of strain P4 and analyzed by TLC using synthetic AHL migration standards and the above mentioned bio-indicator NT1(pZLR4). Strain P4 produced a single AHL molecule spot as judged from the TLC plate, the Rf and shape of which suggested that it might be *N*-(3-hydroxy-octanoyl)-homoserine lactone (3OH,C8-HSL). The presence, in the ethyl acetate extract of the only 3OH,C8-HSL signal molecule, that is not the common signal detected in other *Agrobacterium* strains [33], was confirmed (Fig. 1) using ultra-performance liquid chromatography - tandem mass spectrometer (UPLC-MS/MS) analysis.

### Strain P4 displayed a functional *luxI*-like gene (*cinI*) bordered by two *luxR*-like genes (*cinR* and *cinX*)

Blast analysis of the genome of strain P4 revealed the occurrence of a single *luxI*-like gene that exhibited a 783 bp ORF and encoded a deduced 29.6 kDa protein. This protein consisted of 260 amino acids. The P4 *luxI*-like gene, that we termed *cinI* (see below), was PCR-amplified, cloned into pGEM®-T easy and the production of AHL by the resulting strain was evaluated. The presence of the cloned *cinI* gene in *E. coli* conferred the ability to produce 3OH,C8-HSL.

**Fig. 2:**
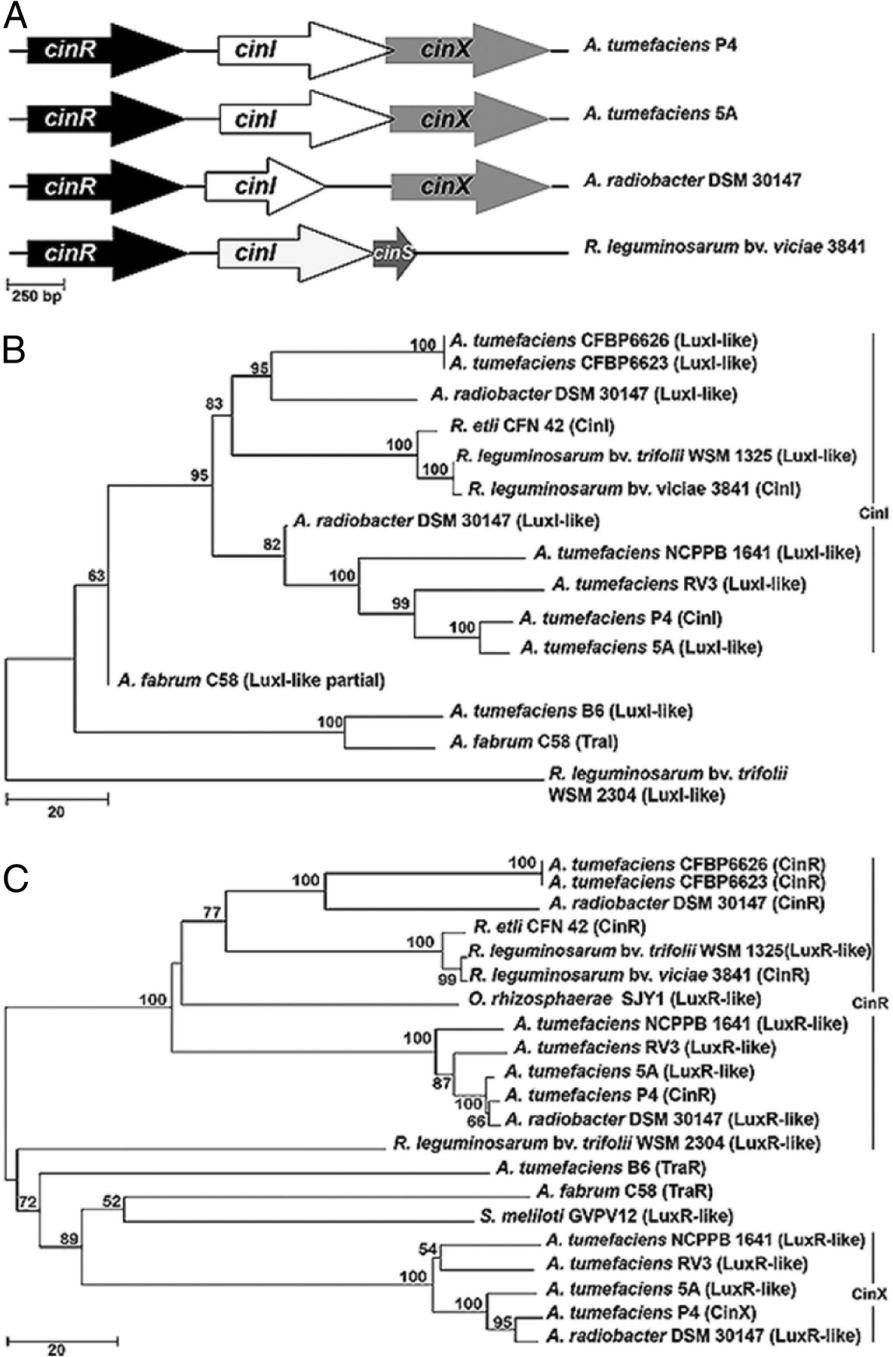
Genetic determinants of the quorum sensing regulation on pAtP4 and related regions of other plasmids. Panel **a**: genetic organization of the *cinI* region on the pAt plasmid of *A. tumefaciens* strains P4 and 5A, *A. radiobacter* DSM 30147 and in the genome of *R. leguminosarum* strain 3841. Gene sizes are drawn to scale (scale at the left bottom part of panel **a**). Panel **b**: Phylogenetic relatedness of the deduced CinI protein of strain P4. The tree has been constructed using the Neighbor-Joining method and bootstrap values calculated from 1,000 re-samplings technique. Panel **c**: Phylogenetic relatedness of the deduced CinR and CinX proteins of strain P4. The tree has been constructed as indicated above

The proteins most related to the P4 LuxI-like protein were other deduced LuxI proteins from four *Agrobacterium* strains, namely *A. tumefaciens* strains 5A, RV3, and NCPPB 1641, and *A. radiobacter* strain DSM 30147 (Fig. 2a, b). The P4 CinI protein was also related to the TraI protein of *Agrobacterium* strain C58 (32 % identity and 45 % similarity). Outside the *Agrobacterium* genus, the closest deduced protein was CinI from *Rhizobium leguminosarum* (57 % identity and 71 % similarity; Fig. 2b), hence the name *cinI* for the P4 *luxI* homologue. It is located on the At plasmid of strain P4 at coordinates 292 921 to 293 703.

The Blast analysis was expanded to the right and left side of *cinI* to search for other possible component(s) of a QS regulatory system as their coding sequences are often located in the vicinity of *luxI*-like genes [42]. This analysis revealed the presence to the left and right regions of *cinI* of two *luxR*-like determinants (AGROTU_05922 and AGROTU_05924; Fig. 2a) that were termed *cinR* and *cinX*, respectively*.* The putative protein CinR (ca. 30 kDa) consisted of 239 amino acids, while the putative CinX protein (27.8 kDa) consisted of 247 amino acids. Both CinR and CinX proteins exhibited the characteristic AHL-binding and DNA-binding domains [43, 44]. The proteins most related to the P4 CinR protein were again the deduced LuxR proteins from *A. radiobacter* DSM 30147 and *A. tumefaciens* 5A, RV3, and NCPPB 1641. Outside the *Agrobacterium* clade, the proteins most related to the P4 CinR protein were those from *Ochrobactrum rhizosphaerae* SJY1 and *Rhizobium etli* with which P4 CinR shared 55 and 54 % identity and 73 and 69 % similarity, respectively (Fig. 2c). The P4 CinR protein was also related to the TraR protein of *Agrobacterium* strain C58 (22 % identity and 44 % similarity). The proteins most related to CinX were the LuxR-like proteins from the above mentioned *Agrobacterium* strains and, outside this clade, CinR from *Sinorhizobium meliloti* GVPV12 with which P4 CinX shared 39 % identity and 59 % similarity (Fig. 2c). CinR and CinX displayed only 29 % identity and 49 % similarity with each other and were therefore only distantly related.

The exact same organization of the *cinR cinI cinX* region was found in data bases only in the four above mentioned *Agrobacterium* strains (*i.e.* 5A, RV3, NCPPB 1641, and DSM 30147; Fig. 2a). A related gene organization was observed in *R. leguminosarum* strain 3841. However in this later strain, *cinX* was absent and replaced by *cinS* [45, 46].

### Transcriptome analysis revealed that *cinI* regulated the expression of genes involved in the transfer of the At plasmid in strain P4

The identification of a *luxI*-like gene involved in the synthesis of an AHL molecule and the presence of *luxR*-like genes that encode proteins with characteristic AHL-binding and DNA-binding domains on pAtP4, are clear indications that they most likely constitute QS regulatory elements. To identify genes potentially regulated by these QS elements, a *cinI* mutant was generated via the insertion of a gentamicin resistance cassette within *cinI* by homologous recombination (Additional file 3). The resulting strain, termed P4cinI, lost the ability to produce any detectable AHL signal, as judged from the analyses of spent culture supernatant and ethyl acetate extracts.

**Fig. 3:**
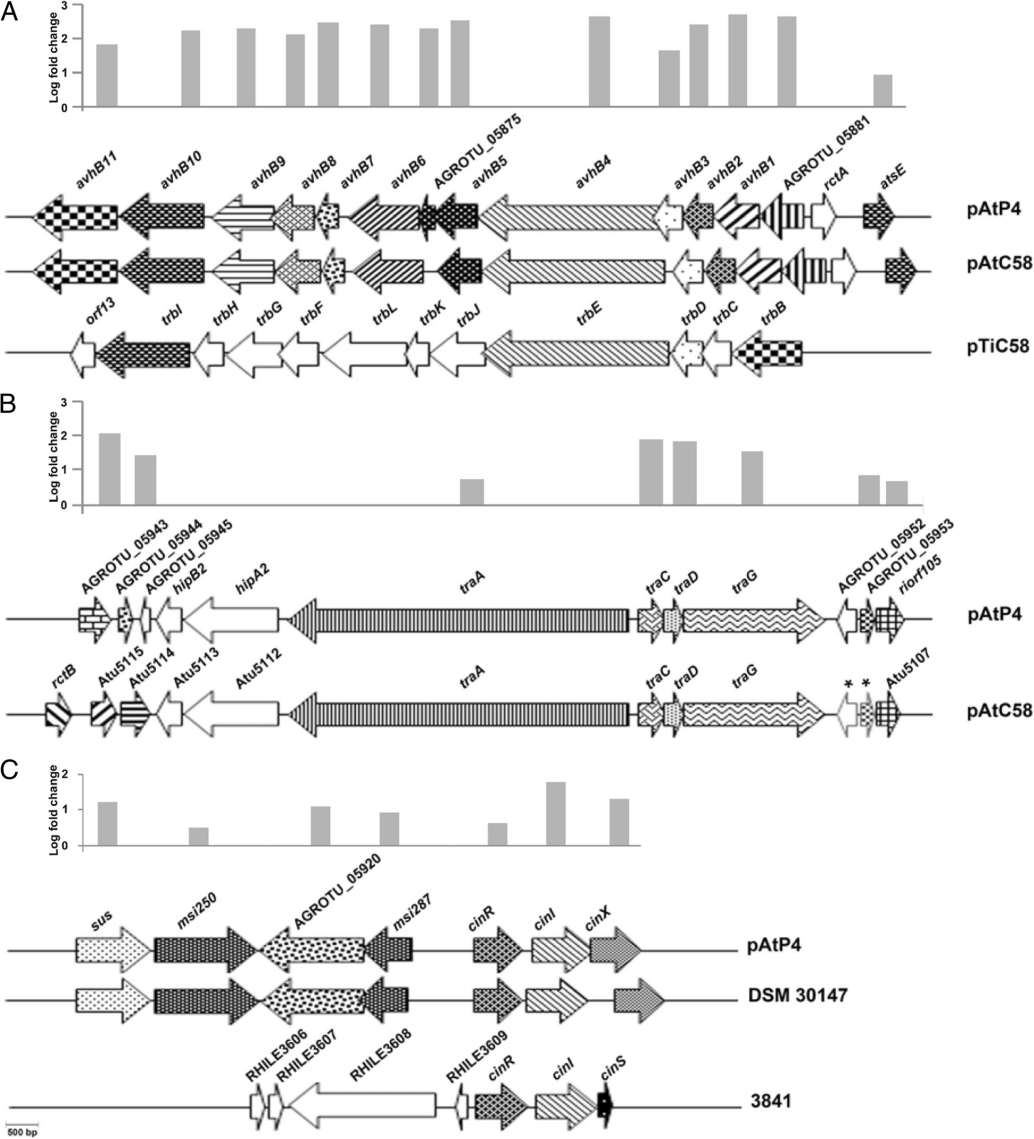
Comparison of three pAtP4 regions displaying upregulated genes*.* Fold change of pAtP4 genes obtained from the transcriptome experiments are shown above each panel. Upregulated genes of the pAtP4 are shown by arrows with different backgrounds while the non-regulated genes are shown by open arrows. Gene sizes are drawn to scale (scale at the *left bottom part* of panel **c**). Panel **a**: the *avhB* cluster of pAtP4 with genes upregulated or not. The *avhB* cluster of pAtP4 was aligned with the *avhB* cluster of pAtC58 and compared with the *trb* cluster of pTiC58. C58 genes displaying significant similarity to *avhB* genes are marked with the same background pattern as used for the corresponding *avhB* gene of pAtP4. Panel **b**: the *tra* cluster of pAtP4 with genes upregulated or not. The *tra* cluster of pAtP4 was aligned with *tra* cluster of pAtC58. Up regulated and non-regulated genes are represented as indicated above. Panel **c**: the *cinR cinI cinX* genomic region with upregulated genes of the pAtP4. The *cinR cinI cinX* genomic region was aligned with the corresponding pAt region of *A. radiobacter* strain DSM 30147 and compared to the one of *R. leguminosarum* bv. *viciae* strain 3841. Up regulated and non-regulated genes are represented as indicated above

RNA was extracted from both P4 and P4cinI strains during the exponential phase of growth, and used to perform transcriptome analyses. Only fold changes above 3 with a p-value lower than 5 10^*−2*^ were considered significant. Using these parameters, 32 differentially expressed genes were identified (Additional file 4), all of them being upregulated in P4 when compared to P4cinI. Most of the genes, *i.e.* 29 out of 32, were located on the At plasmid of strain P4 (Fig. 3). The remaining 3 genes were located on the circular (AGROTU_03028) and linear (AGROTU_05377 and AGROTU_05378) chromosomes. Gene AGROTU_03028 encodes a conserved hypothetical protein, AGROTU_05377 a hypothetical protein, and AGROTU_05378 a putative nucleotidyl transferase.

On pAtP4, a first group of upregulated genes comprised the *avhB* genes plus the genes AGROTU_05875, AGROTU_05881 and *atsE*. This cluster was located between coordinates 241 317 and 252 681 (Fig. 3a). It encompasses another gene, *rctA,* which did not appear to be differentially expressed in strains P4 and P4cinI. The pAt *avhB* genes of strain P4 were orthologs to those of pAtC58 that encode the T4SS_pAt_ involved in the conjugative transfer of this plasmid [26]. AGROTU_05875 encoded a partly deleted *avhB5* gene. The two other genes, AGROTU_05881 and *atsE*, encoded a hypothetical protein and a hypothetical host attachment protein, respectively.

A second group of upregulated genes was located between coordinates 314 321 and 325 085. It comprised the *traA*, *traC*, *traD*, *traG* gene cluster, plus four neighboring genes, namely AGROTU_05943, AGROTU_05944, AGROTU_05953 and *riorf105* (Fig. 3b). The four *tra* genes of strain pAtP4 are orthologs to the *tra* genes of pAtC58. These genes encode components of the DNA transfer and replication (Dtr) system that recognizes and cleaves at the origin of transfer (oriT) of plasmids [26, 47]. The genes AGROTU_05943, AGROTU_05953 and *riorf105* encoded hypothetical proteins with no predicted functional domains, whereas AGROTU_05944 encoded a putative plasmid stabilization protein. Two other genes, namely *hipA2* and *hipB2*, located downstream of *traA* on pAtP4, were orthologous to respectively Atu5112 (*hipA*) and Atu5113 (*hipB* encoding a transcriptional regulator) of pAtC58. These genes were not overexpressed in P4 compared to P4cinI.

The third group of upregulated genes was located between coordinates 285 182 and 294 410 (Fig. 3c). Remarkably, this group of genes encompassed *cinR*, *cinI* and *cinX*, *cinI* being the most upregulated gene within this set of three. The other genes, *sus* and *msi250,* encoded hypothetical proteins, while AGROTU_05920 encoded a hypothetical protein with a metal-dependent hydrolase domain and *msi287* a phosphate regulatory protein.

The upregulation of a subset of 8 of the above mentioned pAtP4 genes (i.e. *avhB11*, *avhB5*, *traA*, *traG*, *cinR*, *cinI, cinX*, and AGROTU_05920) was confirmed in strains P4 and P4cinI by RT-qPCR (Additional file 4).

### CinI and 3OH,C8-HSL regulated the conjugative transfer of the pAt in strain P4

**Fig. 4:**
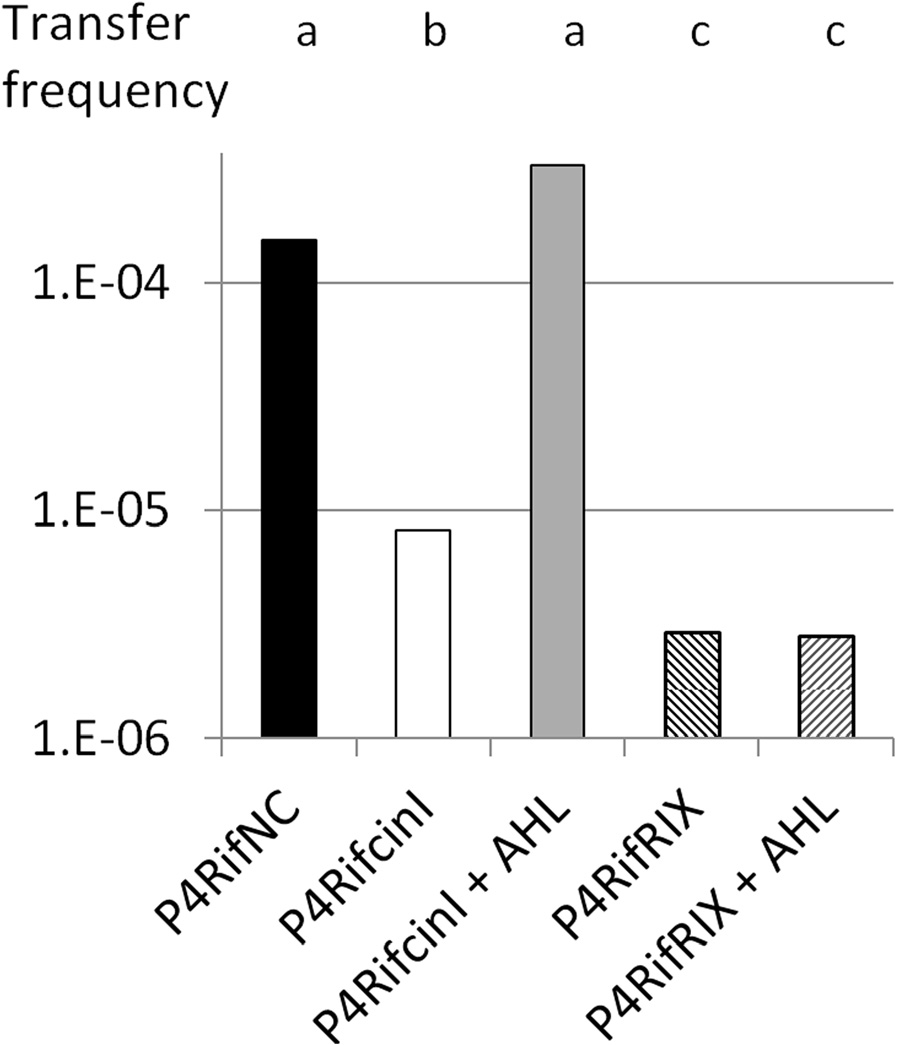
pAt conjugation efficiencies of strain P4 and related mutants. Crosses involved the pAt donor indicated on the x axis and strain C58.00 as a recipient. Conjugation frequencies (y axis) were expressed per recipient cells. A Kruskal-Wallis non parametric test and a post-hoc Tukey test were used to evaluate whether the observed differences were significant (*p* ≤ 0.05) or not (*p* > 0.05). Results are shown by the letters displayed above the graph

The above data strongly support the view that *cinI* and the associated 3OH,C8-HSL molecule controlled the conjugative transfer of the At plasmid of strain P4. To validate this hypothesis, two P4 mutant strains were used as pAt donor strains in conjugation experiments: P4RifcinI that harbored a *cinI* gene disrupted by a gentamicin resistance cassette and P4RifNC that harbored the same gentamicin resistance cassette inserted in a noncoding region of the At plasmid (see Methods). Conjugations were performed with strain C58.00 as a recipient. The average pAt transfer frequencies (expressed per recipient cell) were 1.24 10^−4^ in the cross P4RifNC x C58.00 and 8.9 10^−6^ in the cross P4RifcinI x C58.00. These two values statistically differed (Kruskal-Wallis and post hoc Tuckey; *p* < 0.05; Fig. 4). The transfer frequency of the At plasmid was therefore *ca.* 14 times higher for the strain carrying an intact *cinI* gene than it was for the strain with a mutated *cinI* gene. When 100 nM synthetic 3OH,C8-HSL were added in the media that supported the cross P4RifcinI x C58.00, the conjugation frequency was comparable to that observed in the cross P4RifNC x C58.00 (Kruskal-Wallis and post hoc Tuckey; *p* < 0.05; Fig. 4). These results unambiguously demonstrated that *cinI* and the associated 3OH,C8-HSL signal control the transfer of the At plasmid in strain P4.

The role of CinR and CinX was assessed using another mutant, P4RifRIX that carried a deletion of the *cinR cinI cinX* region replaced by the same gentamicin resistance cassette used to generate P4RifcinI and P4RifNC (see Additional file 3). This mutant was used as a pAt donor strains in conjugation experiments that involved strain C58.00 as a recipient. Crosses were performed either in the absence or in the presence of 100 nM synthetic 3OH,C8-HSL. The average pAt transfer frequencies (expressed per recipient cell) were 2.75 10^−6^ in the cross performed in the absence of the AHL, and 2.91 10^−6^ in the cross performed in the presence of the AHL, two values that do not statistically differ from each other (Kruskal-Wallis and post hoc Tuckey; *p* > 0.05; Fig. 4). These results indicated that either CinR or CinX, or both, are essential for the detection of the AHL signal molecule.

### Quorum sensing regulation of the conjugation of the At plasmid did not confer a selective advantage in tomato plant rhizosphere

To investigate whether the QS regulated transfer of the At plasmid may provide the host bacteria with a selective advantage, a series of experiments that involved various P4 mutant strains were performed. First, the consequences on bacterial growth of the mutation of the P4 gene *cinI* was evaluated *in vitro*, by comparing the generation time of strain P4Rif and P4RifcinI. In AB mannitol, generation times were 2.57 and 2.55 h, respectively. In AB glucose, they were 2.61 and 2.71 h, in LBm 1.74 and 1.96 h, and in TY broth 1.92 and 1.92 h, respectively. These experiments were repeated 5 times independently and the differences observed for each culture media were not significant (Mann and Whitney, *p* > 0.05).

**Fig. 5:**
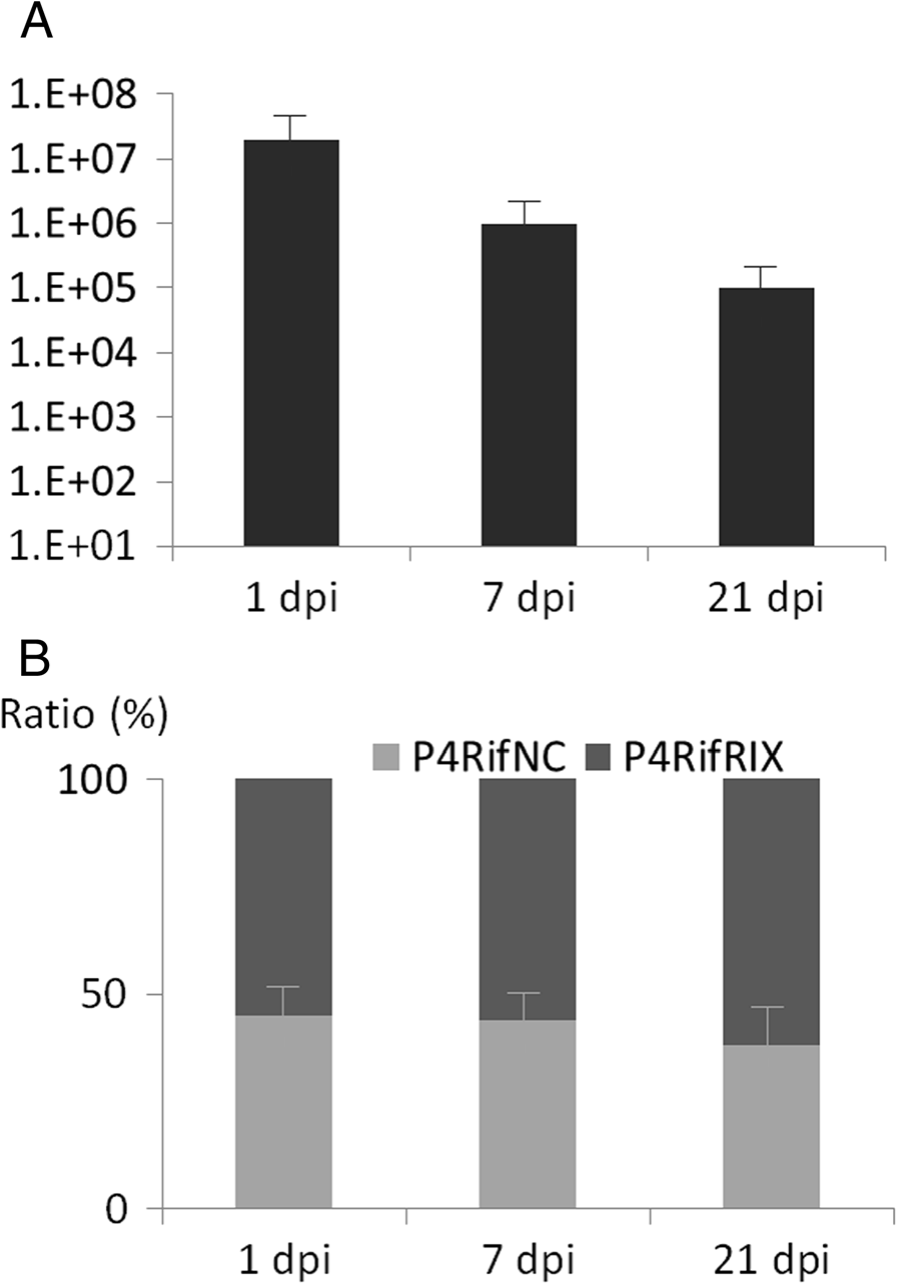
Fitness of P4 and the P4 *cinR cinI cinX* mutant in the tomato rhizosphere. Cell densities (CFU/g of soil) of strains P4RifNC and P4RifRIX inoculated simultaneously on tomato plants cultivated in an unsterilized horticultural soil were assessed by dilution and plating onto the appropriate media (see Methods). Panel **a**: cell density of both co-inoculated P4RifNC and P4RifRIX. Panel **b**: ratio of the cell densities of each strain. At each time point (but not between time points), the values generated by the 3 repeats were statistically analyzed by a Mann and Whitney non parametric test which indicated that the proportion of the cell densities of the two strains were not statistically different (*p*-values > 0.05)

The relative fitness of strain P4 derivatives producing or not 3OH,C8-HSL was next evaluated in a competition experiment in a more ecologically-significant environment. To do so, and because P4 produces large amount of 3OH,C8HSL that can still be sensed by P4RifcinI, we used the *cinR cinI cinX* mutant described earlier. Tomato rhizospheres were inoculated by *Agrobacterium* strains P4RifRIX and P4RifNC at equal cell density and the colonization of the root system assessed at various times by enumeration from soil suspensions of the inoculated bacteria on AB mannitol supplemented by rifampicin and gentamicin. The results (Fig. 5) did not revealed any fitness differences of the two competing strains.

## Discussion

This study led to the identification of the AHL molecule 3OH,C8-HSL produced by the avirulent *Agrobacterium* strain P4. The 3OH,C8-HSL signal is unusual among Agrobacteria but has been described as a QS signal in other systems (e.g. *Burkholderia mallei* [48]). In strain P4, several elements indicate that a QS system controlled the conjugative transfer of pAtP4. These are: (i), the production of 3OH,C8-HSL in P4 culture supernatants, a molecule that belongs to the AHL class of QS signals; (ii), the presence of *cinI*, a *luxI* ortholog in strain P4 involved in the synthesis of the 3OH,C8-HSL signal; (iii), the occurrence of *luxR* orthologs that encoded proteins that possess characteristic AHL- and DNA-binding domains; and, (iv) the results of the transcriptome analysis and conjugation activities. Indeed, from the differential transcriptome analysis of strains P4 and a P4 mutant unable to synthesize the signal molecule, it appears that the wild type P4 strain expressed sets of pAtP4 genes involved in the conjugative transfer of this plasmid at a much higher level than the mutant did. In support of this, the conjugation frequency of the At plasmid of strain P4 was ca. 14 times higher in the wild type strain than it was in the mutant. As a whole, this work is the first identification of the occurrence of QS regulation in the conjugation of an At plasmid in *Agrobacterium*. A possibility exists, however, that a similar system has been identified, but not characterized, by other authors on a transmissible genetic element of *Agrobacterium* [49].

Three sets of genes were upregulated in the wild type strain P4 (when compared with the mutant unable to produce the signal). The first set encoded the T4SS_pAt_ involved in the formation of the conjugation apparatus in the donor bacteria, also termed the mating pair formation (Mpf) complex [50]. The genes and deduced proteins that encode or determine the T4SS_pAt_ of strain P4 were highly related to those present on the At plasmid of strain C58. T4SS are indeed widespread among bacteria, where they ensure the transfer of protein(s) or nucleoprotein complexes outside the bacterial cell (for reviews, see [51, 52]). This is the case of the T4SS of *A. tumefaciens*, known as the Vir system, that promotes the transfer of a single strand copy of T-DNA from the bacteria to the plant cell [53] (for review, see [2]) and that of several plasmid conjugation systems such as those of the above mentioned pAt and pTi in strain C58 (for review, see [52]).

The second set of upregulated genes encoded the DNA transfer and replication (Dtr) system, a protein complex also known as the relaxosome. The relaxosome recognizes and cleaves the *nic* site at the origin of transfer (oriT) of plasmids [26, 47]. The oriT of pAt strain P4 has not been formally identified, but on pAtC58 the origin of transfer was previously located between the *traA* and *traC* genes [26]. Considering the high relatedness of the sequences of *traA*, *traC* and their intergenic region, it is tempting to speculate that the oriT of pAtP4 may be located also here. Based on the similarity of the deduced protein sequences, pAtP4 *traA*, *traC*, *traD* might encode the relaxosome, while *traG* may encode the so-called coupling protein that bridges the relaxosome and its cognate T4SS [54]. In the *Rhizobiaceae*, three Dtr systems have been identified among all species. The types I and II Dtr systems are found to be encoded by autotransferable plasmids, and the type III by mobilizable ones [55]. It has been proposed that class I Dtr systems such as the one encoded by the pTiC58 *tra* genes, are regulated by QS while class II Dtr sytems, including the one encoded by the pAtC58 *tra* genes, are regulated by an RctA repressor associated with an RctB anti-activator [55, 56]. Interestingly, the organization of the four *tra* genes of pAtP4 did not reveal the presence of *traF* and *traB* orthologs downstream of *traA,* a key feature of type I Dtr systems. The absence of *traF* and *traB* orthologs on pAtP4 would therefore place the pAtP4 Dtr system amongst type II systems regulated by RctA, especially considering that a *rctA* ortholog, but no *rctB* ortholog, is present on pAtP4 (Fig. 3). The present study however demonstrated that the pAtP4 type II Dtr system was regulated in a *luxI/luxR* dependent fashion. This finding therefore indicated that type II Dtr systems could also be regulated by QS, contrarily to what has been suggested earlier [55]. We propose that two subclasses of type II Dtr systems exist: a first one regulated by RctA and RctB (e.g. on pAtC58), and a second by RctA and QS (*e.g.* on pAtP4). In agreement with this, a similar situation (presence of the QS genes, presence of the repressor gene *rctA* and absence of the *rctB* gene) was found on the At plasmid of *Agrobacterium* strain 5A (not shown) that could therefore also belong to the second class of Type II Dtr systems.

In relation with the above, genes encoding components of a QS regulatory system have been detected on pAtP4 among those upregulated in the wild type P4 strain with respect to the mutant. The gene cluster structure is quite unusual, as it consisted of a single *luxI*-like gene surrounded by two *luxR*-like genes. This organization appears to be unique in the bacterial world, and found only in *Agrobacterium* strains (P4, 5A, RV3, NCPPB 1641 and DSM 30147). Interestingly, strains P4 and 5A belong to the same *A. tumefaciens* genomospecies 1 and have been isolated in different experiments performed with a soil of the same origin (X. Nesme, personal communication).

Besides the molecular genetics of pAt conjugation, the reported data tackled some ecological aspects of the phenomenon. A recent report indicated that the At plasmid confers a competitive advantage upon the host strain in the rhizosphere but exhibits a high cost under carbon-limiting conditions [22]. Therefore, we investigated whether the ability to transfer the plasmid also favors or hampers bacterial growth in the rhizosphere environment. No fitness differences were observed under the experimental conditions we used between strains P4RifRIX and P4RifNC (Fig. 5). From the above, it is reasonable to conclude that the QS-regulation of the conjugative transfer of pAtP4 did not confer a selective advantage upon the plasmid host in the tomato rhizosphere, at least under our experimental conditions.

## Conclusion

This study led to the identification of novel conjugation system in which QS regulates plasmid transfer. However, contrary to what is observed for the conjugation of the Ti plasmid, the QS regulation of pAt transfer was not stringent (*i.e.* it was not a “on/off switch”), a feature that suggests that additional regulatory components may exist. The ecological and evolutionary relevance of the QS control of plasmid conjugation is still open to debate [21]. Although such a regulatory process might be necessary to prevent a futile mobilization of the donor metabolic resources required for the conjugative transfer of a replicon (that can represent, in the case of the At plasmid, ca. 10 % of its genome) when the donor population is low, QS regulation does not allow the donor to sense whether putative recipients are present. In relation to this, it has been suggested that QS regulation of plasmid conjugation may serve as a way to detect a presence of a biofilm rather than to evaluate a cell density [57]. This suggestion can be related to the diffusion sensing concept described by Redfield [58]. Both hypotheses propose that QS would permit the biological phenomenon, here the conjugative transfer of Ti and At plasmids, to occur only or preferentially in confined environments. Another report suggested that QS regulation of plasmid conjugation may be a way to switch the bacterial behavior from plasmid donor at high cell density to recipient at low cell density [59]. This elegant proposal is based on the existence of genes encoding a plasmid exclusion entry system that relies upon two Trb proteins (TrbJ and TrbK) encoded by the corresponding *trb* genes of Ti plasmids. Interestingly pAtP4, as well as pAtC58, do not harbor *trbJ* and *trbK* orthologs. The ecological relevance of QS regulation of the pAtP4 conjugative transfer therefore remains, to some extent, unclear.

## Additional files

Additional file 1: Map of the genetic determinants involved in Ti plasmid conjugation in *Agrobacterium* strain C58. The functions of operons or genetic regions are as follows: *acc*, agrocinopine catabolism; arc, agrocinopine regulation of conjugation; *noc*, nopaline catabolism; *tra*, conjugative transfer region a; *trb* conjugative transfer region b; vir, virulence. Both the *acc* and *arc* operons are regulated by the repressor AccR. In the presence of agrocinopines A and B or in an *accR* mutant, the *acc* and *arc* operons are expressed. The expression of the *arc* operon permits the production of the QS regulator TraR. The production of the QS signal 3O,C8-HSL by the synthase TraI leads to an accumulation of the signal until its concentration (*i.e.* the cell concentration) is sufficient to activate TraR and allow the expression of the QS-regulated operons *traAFBH*, *traCDG* and *trb*, a phenomenon leading to an increasing production of 3O,C8-HSL and eventually to the transfer of the Ti plasmid. The figure is reprinted from Chan et al. [60] with the kind authorization from the publisher. (PDF 114 kb) Additional file 2: UPLC MS-MS solvent composition. The table provides the composition of the solvent mixture used in the UPLC MS-MS analyses of the concentrated extract of the culture supernatant of strain P4. (DOCX 14 kb) Additional file 3: Mutagenesis of the quorum sensing determinants of pAtP4. All mutations were generated by the insertion of the same Gm resistance cassette that originated from p34S-Gm [39] either at the *Xba*I site of the *cinI* gene or at a reconstituted *Sph*I site generated by the fusion of parts of *cinR* and *cinX* determinants in a synthetic ORF. (PDF 46 kb) Additional file 4: The 32 differentially expressed genes identified by RNA-seq. Only changes above 3 fold with a p-value lower than 5 10^*−2*^ were considered significant. All differentially expressed genes were upregulated in P4 when compared to P4cinI. (PDF 285 kb) Additional file 5: Differentially expressed genes. Xlsx. Four repeats are show for the mutant strain P4cinI (col. B to E) with repetition numbers (e.g. rep1_cinI) and three repeats for the wild type strain P4 (col. F to H), also with repetition numbers (e.g. rep1_P4). Average values are given for the mutant strain P4cinI (col. I) and the wild type strain P4 (col. J). Fold change and Log2 fold change are also shown (col. K and L) along with the raw p values and adjusted p values (col. M and N). (XLSX 15 kb)

## Abbreviations

3O,C8-HSL: *N*-(3-oxo-octanoyl)-L-homoserine lactone
3OH,C8-HSL: *N*-(3-hydroxy-octanoyl)-homoserine lactone
AB: *Agrobacterium* broth
AHL: *N*-acyl homoserine lactone
C8-HSL: *N*-(octanoyl)-L-homoserine lactone
*Ca*.: *Circa* (about)
Dtr: DNA transfer and replication
LBm: Luria Bertani medium, modified
Mpf: Mating pair formation
QS: Quorum sensing
T-DNA: Transferred DNA
TY: Tryptone-yeast medium
UPLC: Ultra-performance liquid chromatography
