# Supplementary material for: Transcriptome analysis revealed that a quorum sensing system regulates the transfer of the pAt megaplasmid in Agrobacterium tumefaciens
Source: BMC Genomics. 2016 Aug 20;17:661. doi: 10.1186/s12864-016-3007-5 (PMC4992315; doi:10.1186/s12864-016-3007-5)

Additional file 1.
Map of the genetic determinants involved in Ti plasmid conjugation in *Agrobacterium* strain C58.

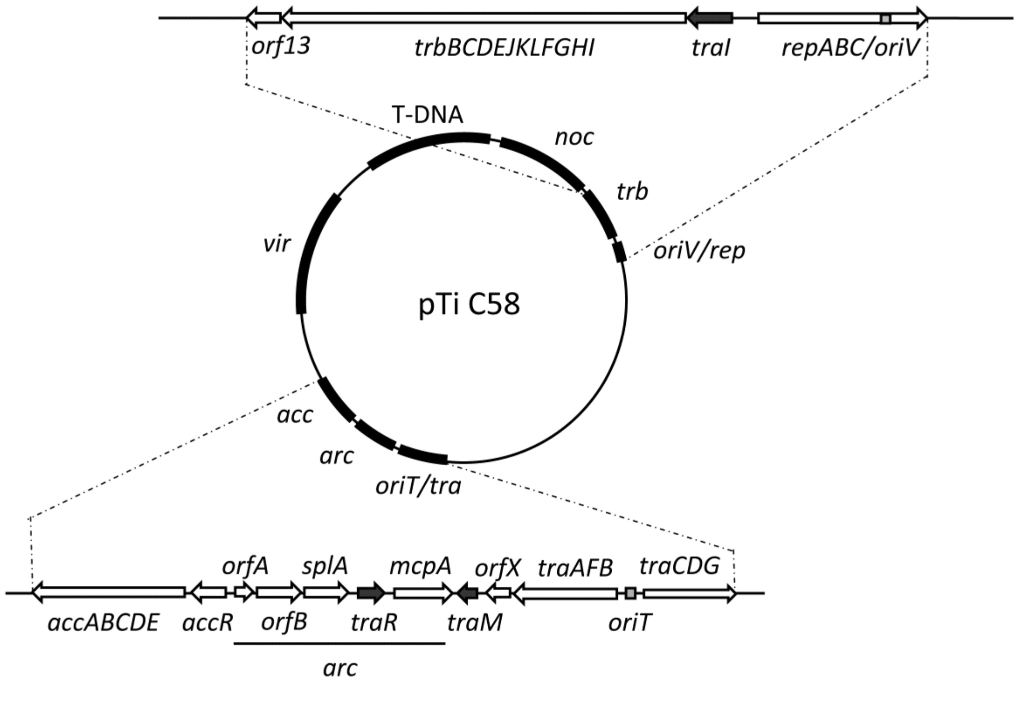

Supplement: Additional file 1: — Map of the genetic determinants involved in Ti plasmid conjugation in Agrobacterium strain C58. The functions of operons or genetic regions are as follows: acc, agrocinopine catabolism; arc, agrocinopine regulation of conjugation; noc, nopaline catabolism; tra, conjugative transfer region a; trb conjugative transfer region b; vir, virulence. Both the acc and arc operons are regulated by the repressor AccR. In the presence of agrocinopines A and B or in an accR mutant, the acc and arc operons are expressed. The expression of the arc operon permits the production of the QS regulator TraR. The production of the QS signal 3O,C8-HSL by the synthase TraI leads to an accumulation of the signal until its concentration (i.e. the cell concentration) is sufficient to activate TraR and allow the expression of the QS-regulated operons traAFBH, traCDG and trb, a phenomenon leading to an increasing production of 3O,C8-HSL and eventually to the transfer of the Ti plasmid. The figure is reprinted from Chan et al. [60] with the kind authorization from the publisher. (PDF 114 kb) [file 12864_2016_3007_MOESM1_ESM.pdf]
