# Supplementary material for: Transcriptome analysis revealed that a quorum sensing system regulates the transfer of the pAt megaplasmid in Agrobacterium tumefaciens
Source: BMC Genomics. 2016 Aug 20;17:661. doi: 10.1186/s12864-016-3007-5 (PMC4992315; doi:10.1186/s12864-016-3007-5)
Supplement: Additional file 2: — UPLC MS-MS solvent composition. The table provides the composition of the solvent mixture used in the UPLC MS-MS analyses of the concentrated extract of the culture supernatant of strain P4. (DOCX 14 kb) [file 12864_2016_3007_MOESM2_ESM.docx]

**Additional file 2.**

**HPLC MS-MS solvent composition for identification of 3OH,C8-HSL.**

| Temps (min) | H_2_O + 0.1% HCOOH | CH_3_CN + 0.1% HCOOH |
| --- | --- | --- |
| 0 | 90 ^a^ | 10 |
| 0.5 | 90 | 10 |
| 5 | 40 | 60 |
| 5.5 | 0 | 100 |

^a^ Values as % of the mixture
