# Supplementary figures and images for: Transcriptome analysis revealed that a quorum sensing system regulates the transfer of the pAt megaplasmid in Agrobacterium tumefaciens
Source: BMC Genomics. 2016 Aug 20;17:661. doi: 10.1186/s12864-016-3007-5 (PMC4992315; doi:10.1186/s12864-016-3007-5)

**Additional file 3.**

**Mutagenesis of the quorum sensing determinants of pAtP4.**

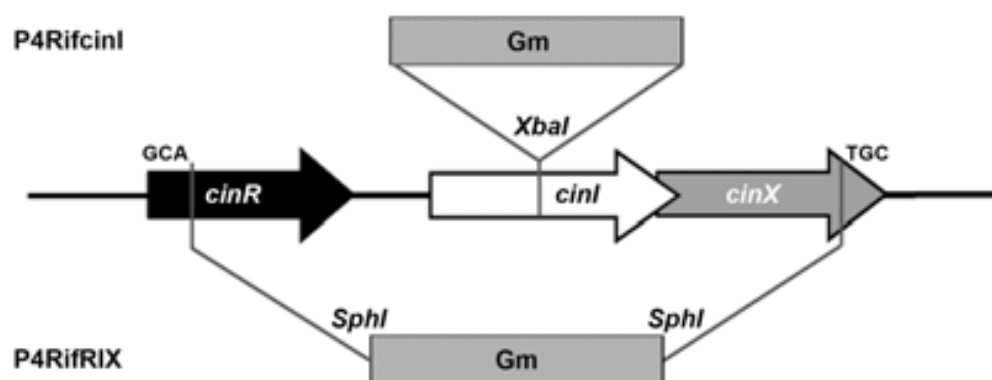

Supplement: Additional file 3: — Mutagenesis of the quorum sensing determinants of pAtP4. All mutations were generated by the insertion of the same Gm resistance cassette that originated from p34S-Gm [39] either at the XbaI site of the cinI gene or at a reconstituted SphI site generated by the fusion of parts of cinR and cinX determinants in a synthetic ORF. (PDF 46 kb) [file 12864_2016_3007_MOESM3_ESM.pdf]
