# Supplementary material for: Transcriptome analysis revealed that a quorum sensing system regulates the transfer of the pAt megaplasmid in Agrobacterium tumefaciens
Source: BMC Genomics. 2016 Aug 20;17:661. doi: 10.1186/s12864-016-3007-5 (PMC4992315; doi:10.1186/s12864-016-3007-5)
Supplement: Additional file 4: — The 32 differentially expressed genes identified by RNA-seq. Only changes above 3 fold with a p-value lower than 5 10−2 were considered significant. All differentially expressed genes were upregulated in P4 when compared to P4cinI. (PDF 285 kb) [file 12864_2016_3007_MOESM4_ESM.pdf]

Additional file 4.

The 32 differentially expressed genes identified by RNA-seq.

Gene name	Fold change [a]	P-value	RT-qPCR Fold change [a]	Closest ortholog Percentage of identity and organism [b]
avhB1	533	2.15 E-116		AGT5Av1_80235 (*avhB1*) Id= 99.71 % *A. tumefaciens* 5A
AGROTU_05881	453	4.36 E-114		between positions 4199088 and 4199627 Id= 99.81% *A. tumefaciens* 5A
avhB4	4521	2.58 E-112		AGT5Av1_80232 (*avhB4*) Id= 99.66 % Id= 99.66 % *A. tumefaciens* 5A
avhB5 [c]	347	3.45 E-103	23 698	AGT5Av1_80231 (*avhB5*) Id= 99.43 % *A. tumefaciens* 5A
avhB7	287	1.19 E-99		AGT5Av1_80228 (*avhB7*) Id= 100 % *A. tumefaciens* 5A
avhB2	257	1.83 E-95		AGT5Av1_80234 (*avhB2*), Id= 99.67 % Id= 100 % *A. tumefaciens* 5A
avhB6	248	1.71 E-91		AGT5Av1_80229 (*avhB6*) Id= 99.89 % Id= 99.89 % *A. tumefaciens* 5A
avhB9	206	1.97 E-89		AGT5Av1_80226 (*avhB9*) Id= 99.06 % Id= 99.06 % *A. tumefaciens* 5A
AGROTU_05875	195	1.78 E-88		AGRT5Av1_80230 Id= 99.57 % *A. tumefaciens* 5A
avhB10	167	1.08 E-85		AGT5Av1_80225 (*avhB10*) Id= 98.72 % *A. tumefaciens* 5A
avhB8	125	3.91 E-82		AGT5Av1_80227 (*avhB8*) Id= 99.85 % *A. tumefaciens* 5A
AGROTU_05943	122	5.81 E-69		between positions 4835062 and 4835412 Id= 97.44 % *A. radiobacter* DSM 30147
traC	81	1.16 E-66		AGT5Av1_80303 (*traC*) Id= 97.57 % *A. tumefaciens* 5A
traD	75	4.38 E-59		AGT5Av1_80304 (*traD*) Id= 99.15 % *A. tumefaciens* 5A
avhB11 [c]	65	5.09 E-58	2 237	AGT5Av1_80224 (*avhB11*) Id= 97.82 % *A. tumefaciens* 5A
cinI [c]	61	1.50 E-43	61 892	AGT5Av1_80284 (*cinI*) Id= 95.91 % *A. tumefaciens* 5A
avhB3	46	1.46 E-34		AGT5Av1_80233 (*avhB3*) Id= 99.71 % *A. tumefaciens* 5A
traG [c]	35	1.31 E-32	3 839	Atu5108 (*traG*) Id= 96.67 % *A. tumefaciens* C58
AGROTU_05944	28	9.01 E-30		between positions 4835488 and 4835622 Id= 100 % *A. radiobacter* DSM 30147
cinX [c]	22	4.14 E-25	143	between positions 4818616 and 4819344 Id= 95.20 %*A. radiobacter* DSM 30147

sus	18	1.08 E-23		ASXYv1_3260077<br>Id= 98.00 % *A. radiobacter* DSM 30147
AGROTU_05920 [c]	13	2.94 E-19	91	between positions 4814075 and 4815474<br>Id= 97.00 % *A. radiobacter* DSM 30147
atsE	9	7.19 E-15		ASXYv1_3260045<br>Id= 98.27 %, *A. radiobacter* DSM 30147
msi287	9	5.34 E-12		AGT5Av1_80281<br>Id= 98.09 % *A. tumefaciens* 5A
AGROTU_05953	7	2.19 E-09		between positions 4845296 and 4845484<br>Id= 98.41 % *A. radiobacter* DSM 30147
traA [c]	6	2.12 E-08	195	AGT5Av1_80302 (*traA*)<br>Id= 97.99 % *A. tumefaciens* 5A
riorf105	5	6.30 E-08		ASXYv1_3260113,<br>Id= 99.71 % *A. radiobacter* DSM 30147
AGROTU_05378	5	2.33 E-07		ATU1Lv1_20006<br>Id= 100 % *Agrobacterium* G1 *foundi*
cinR [c]	4	7.87 E-07	82	ASXYv1_3260081,<br>Id= 98.47 % *A. radiobacter* DSM 30147
msi250	3	2.08 E-05		ASXYv1_3260078<br>Id= 97.61 % *A. radiobacter* DSM 30147
AGROTU_03028	3	2.39 E-05		AGRT5Av1_30210<br>Id= 98.46 % *A. tumefaciens* 5A
AGROTU_05377	3	7.71 E-04		between positions 86915 and 87076<br>Id= 97.53 % *Agrobacterium* G1 *foundi*

a Fold change values have been rounded to the closest whole value and only fold change >3 have been retained in this work

b The identification of the closest ortholog was done by a blastN analysis. The associated information (gene accession, name, and origin) were extracted from MicroScope plateform dedicated for *Agrobacterium* at (https://www.genoscope.cns.fr/agc/microscope/home/index.php)

c The differential expression of these genes has been verified by RT-qPCR using the At plasmid *blcR* gene transcript as a reference
